# Selenium, Mercury, and Their Molar Ratio in Sportfish from Drinking Water Reservoirs

**DOI:** 10.3390/ijerph15091864

**Published:** 2018-08-29

**Authors:** Tara K. B. Johnson, Catherine E. LePrevost, Thomas J. Kwak, W. Gregory Cope

**Affiliations:** 1 Department of Applied Ecology, North Carolina State University, Raleigh, NC 27695, USA; tkjohns2@ncsu.edu (T.K.B.J.); greg_cope@ncsu.edu (W.G.C.); 2 U.S. Geological Survey, North Carolina Cooperative Fish and Wildlife Research Unit, Department of Applied Ecology, North Carolina State University, Raleigh, NC 27695, USA; tkwak@ncsu.edu

**Keywords:** mercury, selenium, selenium:mercury molar ratio, selenium health benefit value, reservoir, bluegill, crappie, largemouth bass, fish consumption, risk assessment

## Abstract

Mercury (Hg) bioaccumulates in aquatic ecosystems and may pose a risk to humans who consume fish. Selenium (Se) has the ability to reduce Hg toxicity, but the current guidance for human consumption of fish is based on Hg concentration alone. The purpose of the present study was to examine the relationship between Se and Hg in freshwater sportfish, for which there is a paucity of existing data. We collected three species of fish from different trophic positions from two drinking water reservoirs in central North Carolina, USA, to assess Hg and Se concentrations in relation to fish total length and to compare two measures of the protective ability of Se, the Se:Hg molar ratio and Se health benefit value (HBV_Se_), to current guidance for Hg. According to the Se:Hg molar ratio, all of the low trophic position fish sampled and the middle trophic position fish sampled from one of the reservoirs were safe for consumption. The same number of fish were considered safe using the HBV_Se_. More fish were deemed unsafe when using the Se:Hg molar ratio and HBV_Se_ than were considered unsafe when using the U.S. Environmental Protection Agency (USEPA) Hg threshold. These findings suggest that the measures of Se protection may be unnecessarily conservative or that the USEPA Hg threshold may not be sufficiently protective of human health, especially the health of sensitive populations like pregnant or nursing mothers and young children. Future examination of the Se:Hg molar ratio and HBV_Se_ from a variety of fish tissue samples would help refine the accuracy of these measures so that they may be appropriately utilized in ecological and human health risk assessment.

## 1. Introduction

Mercury (Hg) is a naturally occurring element that exists in the environment in various states, including elemental, inorganic, and organic. The biogeochemical processes that transition Hg into and out of its various states and transport it throughout the environment can occur naturally, but anthropogenic actions, in particular the burning of coal for electricity, impact these natural processes [1]. Hg is a widespread contaminant in aquatic ecosystems resulting from wide-scale combustion of coal for electricity with variation among water bodies [2] and leading to fish consumption advisories throughout the USA and abroad [3,4]. Hg in the organic form of methylmercury (hereafter Hg) poses the greatest threat to aquatic organisms, such as fish, invertebrates, and waterfowl, and to the humans that consume them. Hg bioaccumulates in fish because of its affinity for tissue proteins; Hg has also been shown to transfer to eggs and correlate with reduced reproductive success, decreased levels of critical sex hormones, and increased embryo and larval mortality [1,5,6]. Dietary exposure to Hg occurs through ingestion of contaminated fish and can ultimately result in bioaccumulation within species and biomagnification up the food web [7]. Neurotoxicity is the greatest concern when Hg accumulates above critical levels in the human body, and Hg can be passed along to developing embryos and breast-feeding infants, which are more sensitive to its neurotoxic effects than adults [8].

Selenium (Se) is another naturally occurring element that is present throughout the environment in various states and is transformed and transported via a complex biogeochemical cycle [9]. Se exists naturally in surface waters at low concentrations but has been identified as a contaminant of interest in some water bodies, primarily those receiving discharge of wastewater from or in close proximity to coal-fired power plants [2,9,10,11,12]. A key difference between Se and Hg is that Se is an essential trace element that is required for the production of selenoproteins involved in tasks such as thyroid and immune function and oxidative defense [13,14]. Some level of exposure to Se is necessary to maintain optimum health, but there is a relatively narrow range between biologically useful levels and toxic levels of Se [15]. Extensive study by Lemly of chronic fish exposure to high levels of Se in water related the exposure to tissue pathology and reproductive impairment, ultimately resulting in individual deaths and, without intervention, a potential for population and ecosystem collapse [10,11]. In birds, Se toxicity (selenosis) from ingestion of contaminated fish has been observed as deformities, decreased hatching rates, and increased egg deaths [1]. In humans, selenosis from chronic Se exposure includes symptoms such as hair and nail loss, skin lesions, and nervous system impairment, though these symptoms occur at much higher exposure levels than those documented from fish consumption [15].

Potential exposure to contaminants from fish consumption can pose a risk to human health, given the known contaminant load among fish and wildlife species. Human populations that tend to be especially sensitive to the toxic effects of Hg include young women (future mothers), expecting mothers, nursing infants, young children, and subsistence consumers [8,16]. However, nutritional and health benefits linked to fish consumption (in particular, benefits related to omega-3 polyunsaturated fatty acids) as part of a balanced diet include decreased risk of coronary heart disease, kidney protection, infection resistance, and others [15,17]. A meta-analysis by Mozaffarian and Rimm found that, generally, the benefits of some fish consumption outweigh the potential risks inherent with contaminants such as Hg [18]. Furthermore, consumption of modest concentrations of Se in fish is associated with strong immune response, optimum thyroid function, antioxidant action, possible cancer suppression, reduced risk of certain heart diseases, and, most relevant to fish consumption, a potentially protective effect against Hg toxicity [15]. 

Regulatory guidelines for water quality and recommendations for freshwater fish consumption exist at local, state, and national levels in the USA to protect humans, especially the aforementioned sensitive populations, from potential hazards related to the ingestion of Hg and other contaminants [3,19,20,21,22]. Fish consumption advisories are issued by federal, state, local, or tribal agencies and are typically broad, providing recommendations by water body, species, and/or population subgroup (see, for example, Table 1) [3,22]. In the USA, Hg contamination is responsible for most fish consumption advisories [3]. To avoid unnecessarily eliminating freshwater fish as a nutritious food source, it is necessary for fish consumption advisories to be as accurate as possible. Therefore, it is pertinent to determine the extent to which Se might protect against Hg toxicity so that advisories for fish contaminated with Hg are appropriately protective.

An experiment published by Ganther et al. in 1972 was among the first to demonstrate that Se and Hg in fish tissue at a molar ratio of >1:1 had the potential to reduce the absorption of Hg [24]. Other investigators have since examined the Se:Hg molar ratio in fish [25,26,27,28,29,30], with most attempting to determine the precise protectiveness, and thereby the consumption implications, of the ratio for human health. Though the potential for Se to somewhat mitigate the neurotoxic effects of Hg is well established, the extent to which it does so, as evaluated by the Se:Hg molar ratio, is otherwise highly variable among species, geographic regions, and water bodies [25,27,28]. A recent review by Eagles-Smith et al. identified a lack of consensus regarding the use of the Se:Hg molar ratio and whether Se is universally protective against Hg toxicity or only for some mechanisms, such as neurotoxicity [31]. The authors emphasized the need for further study, and epidemiological research in particular, before the ratio can be implemented in environmental and human health risk assessment. Other authors have noted a lack of data for this ratio in freshwater fish [17,32,33,34], which is important for the development of advisories, because freshwater fish tend to contain much lower concentrations of Se than saltwater fish [13]. One recent study of the molar ratio in freshwater fish from the Brazilian Amazon indicated that carnivorous fish were unsafe for consumption, while iliophagous and herbivorous fish were safe [35]. With continued study of the molar ratio in freshwater fish, it may become possible to extend generalizations such as this to development of fish consumption recommendations.

Another more recent measure of protection based on Se and Hg levels in fish is the Se health benefit value (HBV_Se_), proposed by Ralston et al. and defined as: HBV_Se_ = ( [Se – Hg] / Se) × (Se + Hg), where Se and Hg are molar amounts [32]. The HBV_Se_ calculation takes into account the body’s biochemical use of Se, which is required for tasks such as the production of selenoproteins involved in various beneficial biological functions [13,14]. A HBV_Se_ value >0 suggests the presence of Se in excess with respect to the body’s biochemical demand and is, therefore, considered protective; a value <0 is considered risky, indicating that inhibition or sequestration of Hg by Se is unlikely. The measure was originally developed to examine in utero exposure from maternal consumption of seafood, but has since been applied more broadly as a means to examine the Se:Hg ratio and to support the development of better fish consumption advisories [28,33,34]. For example, the HBV_Se_ calculated for freshwater fish of the Upper Great Lakes region, USA, suggested that, in general, wild-caught fish were safer than farmed fish because of higher Se [36]. The study authors reported that incorporating an indicator of the nutritional benefit of fish consumption, such as the HBV_Se_, may improve risk communication by simultaneously informing consumers of the risks and benefits of fish consumption.

To add to the body of data regarding freshwater fish, metal concentrations, and the Se:Hg molar ratio, we measured Hg and Se concentrations in muscle tissue of three common sportfish species of varying sizes in two drinking water reservoirs in central North Carolina, USA. We then examined the relationships among contaminant measures, fish species and size, and water body. The molar ratio of Se to Hg in freshwater fish tissue from this region has not previously been explored [33]. Furthermore, the water bodies in this study were of particular interest because drinking water reservoirs tend to show greater fluctuation in hydroperiod (i.e., water level) than other freshwater bodies, leading to higher Hg concentrations in fish after periods of high abstraction and drawdown for water consumption [37,38,39,40]. These two specific water bodies also support popular recreational fisheries with fish consumption advisories in effect. An improved understanding of Hg, Se, their molar ratio, and the HBV_Se_ as functions of fish total length informs risk assessment and communication related to fish consumption.

## 2. Materials and Methods 

### 2.1. Site Selection and Sampling

**Figure 1:**
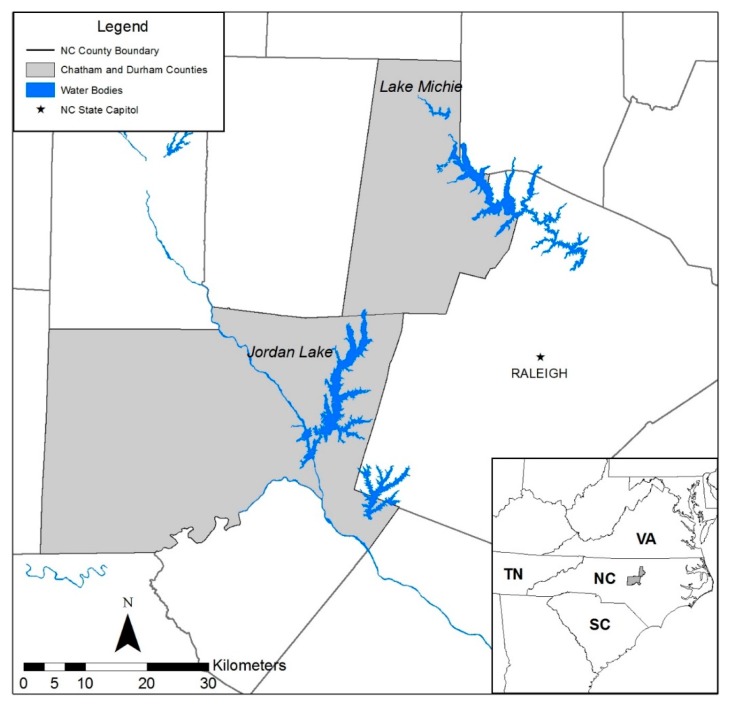
Map depicting the study sites, Jordan Lake and Lake Michie in North Carolina, USA.

We sampled fish in two different years (2014 and 2016) from two drinking water reservoirs, B. Everett Jordan Lake (Jordan Lake) and Lake Michie, in central North Carolina, USA (Figure 1). Jordan Lake is located mostly within Chatham County, North Carolina, covers 5625 ha, and contains a volume of approximately 1.73 × 10^8^ m^3^ at its normal 104-m mean sea level (MSL) elevation [41]. After the U.S. Army Corps of Engineers determined a need for a water resource to provide drinking water and manage flooding, construction of the dam began in 1967, and Jordan Lake was completed in 1982 [41]. The Jordan Lake reservoir provides drinking water to municipalities within the counties of Chatham, Durham, Orange, and Wake in North Carolina and serves as a popular location for recreational fishing [42]. Lake Michie was completed in 1926 to provide water supply for the City of Durham; it is located within northern Durham County, North Carolina [43]. The lake covers 206 ha, contains a volume of 1.365 × 10^7^ m^3^ and has a 104-m MSL elevation when it is full [44,45], and currently maintains 249 days of drinking water with an average delivery of 89 × 10^6^ L/day [46]. Lake Michie is also a popular recreational fishing location that openly advertises its excellent largemouth bass population [47]. 

To sample species representative of different trophic positions, as suggested by Hall et al. [48], and those commonly consumed by recreational and subsistence fishers, as documented by the North Carolina Wildlife Resources Commission [49], we collected bluegill (*Lepomis macrochirus*), black and white crappie (*Pomoxis nigromaculatus* and *Pomoxis annularis*, collectively referred to as “crappie” hereafter), and largemouth bass (*Micropterus salmoides*), 15 fish of each species from each lake via boat-mounted, pulsed-direct current (DC) electrofishing. Bluegill were selected as the representative low trophic position species, based on their diet of insects, zooplankton, and plant material [50]. Crappie were considered an intermediate trophic position species because they are insectivores early in life and become piscivorous as they grow larger, adding small fish, and occasionally invertebrates, to their diet [51]. Largemouth bass represented the high trophic position species because, as apex predators, they consume a diet largely consisting of other fish, and occasionally benthic invertebrates [52].

We classified each fish into one of three size categories based on total length, with an effort to collect five fish in each size class; these quantities were previously supported by Sackett et al. [2]. Based on North Carolina regulations [53], a small fish was categorized as being below the typical fishery harvest length limit, except for bluegill, which do not have a length limit; a medium fish was within an intermediate size range for harvest and consumption; and a large fish was one typically considered a quality catch (see Table 2 for lengths). Fish were collected between 11 April and 11 June 2014, and 20 April and 8 June 2016. In some instances, it was not possible to collect five fish of each size per species. The following quantities and sizes of fish were collected when the target of five could not be achieved: 4 small crappie (Jordan Lake, 2014), 0 large crappie and 4 medium largemouth bass (Lake Michie, 2014), 3 large crappie and 4 medium largemouth bass (Jordan Lake, 2016), and 2 large bluegill and 4 large crappie (Lake Michie, 2016).

### 2.2. Fish Tissue Analysis

Fish were collected by boat-mounted, pulsed-DC electrofishing with efforts to retain the three size classes of the same species from each lake, as above. Fish were euthanized by immediate placement into an ice-water slurry to induce temperature shock according to North Carolina State University-approved protocols (IACUC 15-042-O). Total length (mm) and wet weight (g) were recorded for all fish. All samples were stored frozen at −20 °C until further processing.

Using standard U.S. Environmental Protection Agency (USEPA) fish processing protocols [54], muscle tissue samples were dissected and homogenized. Following homogenization, the samples were frozen at −80 °C until further processing. In preparation for contaminant analyses, muscle tissue samples were lyophilized and manually homogenized. RTI International (Research Triangle Park, North Carolina) analyzed the fish tissue samples for Hg (2014 and 2016 samples) and Se (2016 samples only). As it has been established that >95% of Hg within fish is methylmercury [55], total Hg concentration in tissue samples was determined using a Milestone DMA-80 direct Hg analyzer according to USEPA method 7473 [56]. Se was analyzed with a modified version of Method 3050B [57] and a Thermo X-Series II ICP-MS or a Thermo iCAP6500 ICP-OES, depending on the concentration of the analyte present in the sample.

Each batch of tissue samples analyzed was accompanied by appropriate quality assurance protocols, including blanks, Hg- or Se-spiked samples, duplicates, and National Institute of Standards and Technology (NIST) certified reference material (2976 muscle tissue). The recoveries of certified reference material ranged from 93 to 111%, with an overall mean recovery of 101.3 ± 4.8% standard deviation. Quality control was also conducted by separately analyzing duplicates (10%) of our freeze-dried tissue samples. All blanks, spiked samples, and duplicates were within acceptable limits.

### 2.3. Statistical Analyses

Statistical analyses were performed with JMP, version 13.0.0 [58]. Hg data were combined for 2014 and 2016 after a preliminary least squares analysis of Hg concentration by year revealed no statistical difference between the two years. The effect of fish total length on measured fish tissue Hg, fish tissue Se, and Se:Hg molar ratio was evaluated by plotting the results by species and lake. We elected to analyze the effect of total length on contaminant values based on previous research on the effect of length on tissue Hg [7] and because previous studies of the Se:Hg molar ratio have produced highly variable results when the ratio was plotted as a function of Hg concentration [25,27,29,33]. The data generally displayed an exponential trend with increasing length. We log_e_-transformed all tissue concentration data to comply with the normal distribution assumption and performed a separate one-way analysis of variance for each contaminant and the Se:Hg ratio (ANOVA; α = 0.05) to determine the significance of total length. Se health benefit values (HBV_Se_) were calculated according to Ralston et al. [32].

## 3. Results

### 3.1. Mercury

**Figure 2:**
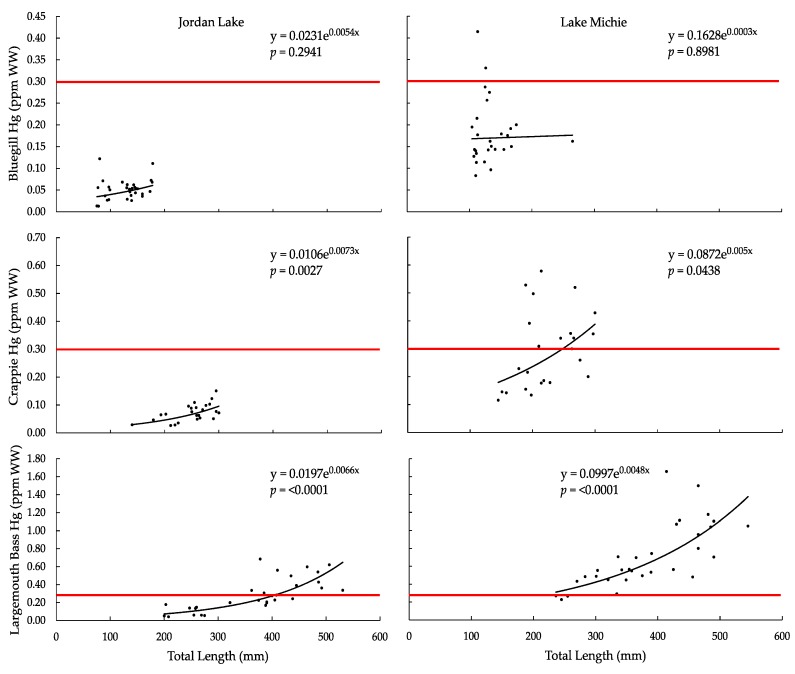
Tissue Hg concentration (ppm WW) vs. total length (mm) in (**a**) bluegill from Jordan Lake, (**b**) bluegill from Lake Michie, (**c**) crappie from Jordan Lake, (**d**) crappie from Lake Michie, (**e**) largemouth bass from Jordan Lake, and (**f**) largemouth bass from Lake Michie. The red horizontal line indicates the U.S. Environmental Protection Agency 0.3-ppm Fish Tissue Residue Criterion.

For each species in both reservoirs, Hg concentrations (ppm wet weight; WW) increased exponentially with total length (Figure 2). The trend was more pronounced from bluegill to crappie to largemouth bass, with the relationship being statistically significant for crappie and largemouth bass (*p* < 0.05; Table 3). The increase in Hg concentration appeared to reflect the increase in total length from one species to the next and within each species, and supported the previous finding that length is correlated with Hg concentration [7]. For each species, Hg concentrations were greater in fish from Lake Michie than in fish from Jordan Lake.

The U.S. Food and Drug Administration action level for Hg in fish is 1.0 ppm [21], and none of the fish sampled from Jordan Lake exceeded this action level; however, 8 of 31 largemouth bass from Lake Michie had Hg concentrations that exceeded the action level. In North Carolina, USA, at the time of fish collection, the Department of Health and Human Services considered 0.4 ppm an action level for Hg fish advisories [23]; since 2017, a risk-based screening process has been implemented by the Department [59]. From Jordan Lake, no bluegill or crappie contained concentrations in excess of that value, but 7 of 31 largemouth bass had Hg concentrations greater than 0.4 ppm. From Lake Michie, 1 of 27 bluegill, 5 of 24 crappie, and 27 of 31 largemouth bass exceeded the North Carolina 0.4-ppm action level. The USEPA Fish Residue Criterion is 0.3 ppm for methylmercury [20]. The number of individuals exceeding the USEPA criterion was similar to that exceeding the North Carolina action level: 0 bluegill, 0 crappie, and 12 of 31 largemouth bass from Jordan Lake; and 2 of 27 bluegill, 11 of 24 crappie, and 27 of 31 largemouth bass from Lake Michie.

### 3.2. Selenium

**Figure 3:**
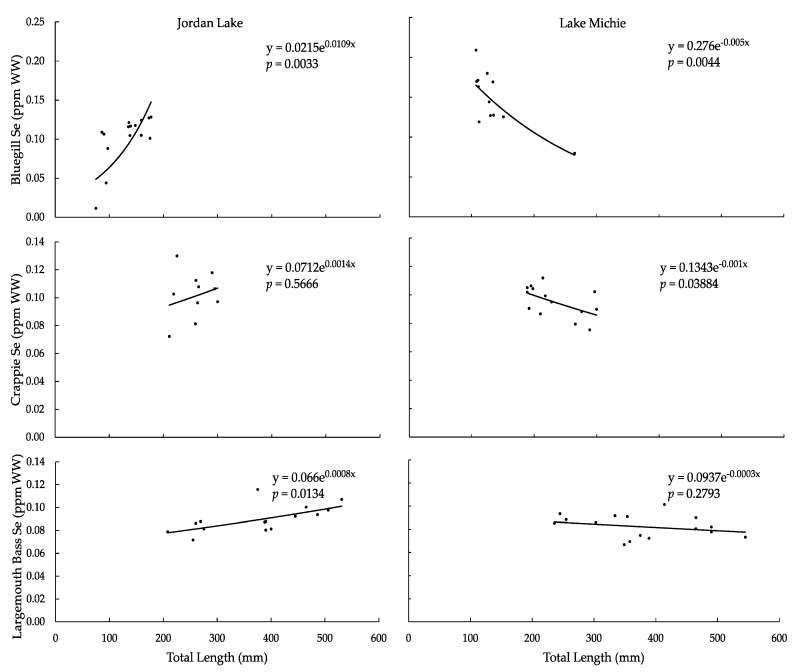
Tissue Se concentration (ppm WW) vs. total length (mm) in (**a**) bluegill from Jordan Lake, (**b**) bluegill from Lake Michie, (**c**) crappie from Jordan Lake, (**d**) crappie from Lake Michie, (**e**) largemouth bass from Jordan Lake, and (**f**) largemouth bass from Lake Michie.

For all species in Jordan Lake, Se concentrations increased with total length; in Lake Michie, the concentrations decreased with length (Figure 3). The relationship was significant for bluegill and largemouth bass in Jordan Lake, and for bluegill and crappie in Lake Michie (*p* < 0.05; Table 3). Mean Se concentrations and ranges for all species in Jordan Lake were similar, despite the differences in lengths, while the mean concentration decreased and the range narrowed from bluegill to crappie to largemouth bass in Lake Michie (Table 3). Bluegill in Lake Michie had the highest mean and range of Se concentrations of all species groups in either reservoir.

In 2016, the USEPA published an updated Se criterion for freshwater and for muscle tissue and egg-ovary tissue in freshwater fish; the level relevant to our study is 11.3 ppm dry weight (DW) for fish muscle tissue [60]. Using the general assumption that fish tissue is approximately 75% water [61], the USEPA Se criterion is approximately 2.8 ppm WW. None of the fish we sampled from either reservoir exceeded the 2016 USEPA criterion, or the North Carolina pre-2017 action level of >10 ppm WW [23], for Se in fish muscle tissue.

### 3.3. Selenium:Mercury Molar Ratio

**Figure 4:**
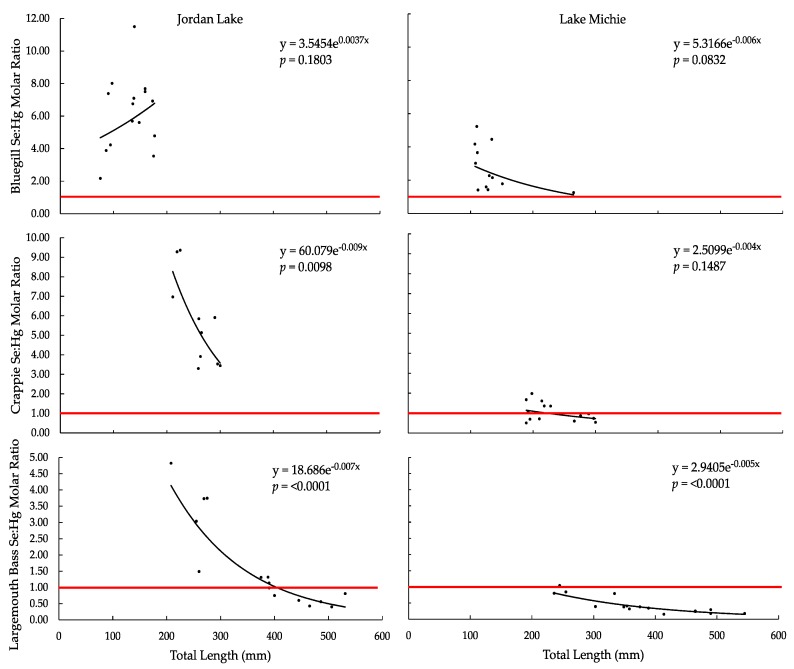
Tissue Se:Hg molar ratio vs. total length (mm) in (**a**) bluegill from Jordan Lake, (**b**) bluegill from Lake Michie, (**c**) crappie from Jordan Lake, (**d**) crappie from Lake Michie, (**e**) largemouth bass from Jordan Lake, and (**f**) largemouth bass from Lake Michie. The horizontal red line indicates the 1:1 molar ratio threshold.

The Se:Hg molar ratio decreased exponentially with increasing total length for all species in both lakes, except for bluegill in Jordan Lake (Figure 4). For both lakes, the range of ratio values was the largest in bluegill, followed by crappie, and then largemouth bass; the mean ratios followed the same trend (Table 3). The Se:Hg ratio was significantly correlated with total length in crappie from Jordan Lake only (*p* < 0.05) and in largemouth bass from both reservoirs (*p* < 0.0001; Table 3). For all species, the mean ratio in Jordan Lake fish exceeded the proposed protective value of >1:1 [24], though some individual largemouth bass (7 of 15) had ratios <1:1. The mean ratio in Lake Michie was >1:1 for bluegill and crappie but not for largemouth bass; additionally, some individual crappie (8 of 14) and all but one individual largemouth bass (15 of 16) had ratios <1:1.

### 3.4. Selenium Health Benefit Values

The HBV_Se_ values (Table 4) were positive for all bluegill in both reservoirs and for all crappie in Jordan Lake; the mean values for these fish were 1.24–1.46. Several crappie in Lake Michie (8 of 14) and a portion of largemouth bass in both reservoirs (7 of 15 from Jordan Lake, and 15 of 16 from Lake Michie) had negative HBV_Se_ values. The means for these fish were between −1 and 0, except for largemouth bass in Lake Michie, for which the mean value was −12.03.

## 4. Discussion

### 4.1. Mercury

We found that Hg concentration increased with total length (*p* < 0.05 for crappie and largemouth bass in both reservoirs) and that many more fish in Lake Michie exceeded the USEPA fish residue criterion than in Jordan Lake. With the USEPA Hg criterion under consideration, it would follow that fish consumption advisories may be warranted for crappie and largemouth bass at Lake Michie and for largemouth bass at Jordan Lake. A statewide fish consumption advisory for Hg in largemouth bass is already in effect, recommending that sensitive populations (i.e., women of childbearing age, pregnant or nursing women, and children under 15 years of age) not consume largemouth bass caught anywhere in North Carolina, USA, and that all others consume no more than one 170-g (6-ounce) meal per week [62]. The same advisory is in effect for black crappie but only from North Carolina waters south and east of Interstate-95, a landmark identifying the lower-pH waters in the eastern part of the state where fish Hg concentrations tend to be greater [22,63]. Crappie from Lake Michie do not fall under this advisory. Creel limits for these species further complicate the risk communication process. In the interest of fishery management, up to 5 largemouth bass may be caught per day from either reservoir with a minimum length of 356 mm (though 2 fish may be harvested under this length), and up to 20 black or white crappie may be harvested per day from Jordan Lake with a minimum length of 254 mm [53]. There are no creel limits for crappie in Lake Michie or for bluegill in either reservoir. Although creel limits promote fish and ecosystem health and equitable harvest, they may inadvertently promote consumption of fish with higher levels of Hg contamination [7], especially by individuals who do not fully understand the relationship between fish length and Hg concentration. 

Regarding the health of fish and other wildlife, previous studies have reported Hg concentrations in various organisms and their tissues that have the potential to induce toxic effects. In rainbow trout (*Oncorhynchus mykiss*) and fathead minnow (*Pimephales promelas*) eggs, a concentration of 0.07–0.10 ppm Hg has been linked to increased embryo mortality, that of 0.5 ppm to inhibited larval survival and reproduction, and that of 0.01–0.63 ppm to adult fish with decreased fertilization success [5]. In fathead minnows, concentrations of 0.87 and 3.93 ppm Hg DW (0.22 and 0.98 ppm WW, respectively) have been associated with decreased sex hormones in males and females and decreased spawning success [6]. Dietary levels of 0.1 ppm Hg for sensitive mammals, 0.2–0.5 ppm for sensitive birds, and 11 ppm for other mammals have been correlated with various negative effects [1]. We did not collect egg Hg data in this study, but Hg has previously been observed in largemouth bass eggs from lakes in eastern North Carolina at levels higher than the USEPA fish residue criterion [5]. With mean tissue Hg concentrations (across all species) ranging from 0.01 to 0.68 ppm from Jordan Lake and from 0.08 to 1.66 ppm from Lake Michie, not only does Hg pose a human health risk from fish consumption, but the fish themselves and other piscivorous organisms within the ecosystems may also be at risk.

Many biotic and abiotic factors can affect tissue Hg concentrations within a water body. Research by Sackett et al. [64,65] determined that Hg accumulation within a water body is primarily a function of fish species, fish trophic status, ecoregion, and water pH, and that Hg concentration within a species is positively correlated with fish length [7]. Additionally, fish from water bodies near (<10 km) coal-fired power plants have lower Hg and higher Se concentrations in tissues than those from water bodies far (>30 km) from coal-fired power plants [2]. Other factors such as fish age, season, and hydroperiod may also affect Hg accumulation [40]. Species, trophic status, ecoregion, fish size groups (length), and season were similar between the two reservoirs we sampled. Additionally, the pH values were similar, ranging 7.8–8.8 at Lake Michie in 2015 [66] and 6.8–9.4 at Jordan Lake in 2016 [67]. Both reservoirs are >30 km from the nearest coal-fired power plant, suggesting that proximity to power plants may not fully explain the elevated tissue Hg in fish from Lake Michie compared to Jordan Lake. Fish age was not examined in this study; therefore, it is not possible to determine if it played a role in the measured Hg concentrations. Though both water bodies are drinking water reservoirs, and thus are subject to fluctuations in water level relative to weather and drinking water demand, Jordan Lake is larger and has many more inflowing tributaries than Lake Michie. Thus, it is also possible that the higher Hg concentrations in Lake Michie fish are influenced by a more pronounced hydroperiod [37,38,39,40].

### 4.2. Selenium

All species in Jordan Lake exhibited a positive relationship between tissue Se concentration and total length (*p <* 0.05 for bluegill and largemouth bass), but all species in Lake Michie exhibited a negative correlation (*p <* 0.05 for bluegill and crappie). Although the intraspecific trend in tissue Se with increasing length was opposite in the two reservoirs, the overall mean tissue Se concentrations and ranges were comparable for all species in both reservoirs. Thus, it is possible that the negative relationship between tissue Se and length in Lake Michie is related to the higher concentrations of Hg in fish from that reservoir. A recent study by Wang and Wang using black seabream (*Acanthopagrus schlegeli*) demonstrated that Se increased demethylation of organic Hg, converting it to inorganic Hg, and that the process primarily occurred within the intestine of that species [68]. Based on those findings, one possible explanation for the trend in Lake Michie is that Se was being utilized for Hg demethylation in the intestine and was, therefore, present at lower levels in the tissue of larger (and higher Hg) fish. Other studies suggest that the presence of Se results in the formation of Se–Hg complexes (decreasing the bioavailability of Hg) or increases the elimination of Hg [13,69,70,71,72]. Thus, it is also possible that Se–Hg complexation was occurring within other organs, reducing the concentration of detectable tissue Se, or that tissue Se was lower because of its role in the elimination of Hg in Lake Michie fish. A precise explanation for the opposite tissue Se trend in fish from the two reservoirs is not possible without further investigation of these parameters.

The 2016 USEPA Se criterion for the protection of human health is approximately 2.8 ppm WW (converted from 11.3 ppm DW) [60]. None of the fish we sampled from either reservoir exceeded the criterion for Se in fish muscle tissue, suggesting that humans can safely consume these fish without experiencing selenosis. Studies of Se toxicity in fish and other members of aquatic ecosystems have revealed additional toxic thresholds. For bluegill, toxicity can be observed at levels greater than 12 ppm DW (3 ppm WW) in the liver, 10 ppm DW (2.5 ppm WW) in eggs, 8 ppm DW (2 ppm WW) in muscle, and 4 ppm DW (1 ppm WW) in the whole body [11]. For fish in general, toxicity can occur when egg Se concentrations are ≥10 ppm or when surrounding water Se concentrations are 5–10 ppm [10]. Decreased hatchability and deformed embryos have been observed in birds with egg Se concentrations of 3 ppb [1]. Based on our findings, fish and other organisms within the surrounding aquatic ecosystems are not likely to experience toxic effects of Se.

### 4.3. Selenium:Merucry Molar Ratio

Consistent with previous studies of the Se:Hg molar ratio in freshwater fish [25,26,28,29,33], we found that our data exhibited broad interspecific and intraspecific variation. Overall, the Se:Hg molar ratio decreased with increased length in all species in both reservoirs, except for bluegill in Jordan Lake. As Se means and ranges were similar in all species and reservoirs (but slightly higher for bluegill in Lake Michie), the increase in Hg from bluegill to crappie to largemouth bass in each reservoir was the driver behind the decrease in the Se:Hg ratio among those species.

There is no current guidance threshold or use for the Se:Hg ratio because of its high variability, but as it is further examined and understood, it may become useful in human health and environmental risk assessment of Hg toxicity [14]. Despite this variability, the discussion in current research (e.g., [28,29,31,33]) often still revolves around the ratio proposed by Ganther et al., whereby a Se:Hg molar ratio >1:1 confers protection to even the most sensitive populations, while a value <1:1 does not [24]. Using that threshold, ratios from our samples were protective for all bluegill; for all crappie from Jordan Lake and for 6 of 14 crappie from Lake Michie; and for 8 of 15 largemouth bass from Jordan Lake and 1 of 16 largemouth bass from Lake Michie. The Hg concentrations made the distinction between the higher (i.e., more protective) ratios from Jordan Lake and the lower ones from Lake Michie, as Se concentrations were similar in the two reservoirs.

In comparing the number of individual fish that had tissue Hg concentrations above the 0.3-ppm USEPA criterion (unsafe for consumption, especially for sensitive subpopulations) to the number of individuals that had ratios <1:1 (proposed level of insufficient Se for protection of developing embryos against Hg toxicity), the number of individuals with molar ratios <1:1 was greater. Five largemouth bass from Jordan Lake had Hg concentrations >0.3 ppm, while 7 had a Se:Hg molar ratio <1:1. From Lake Michie, 6 crappie versus 8 crappie and 12 largemouth bass versus 15 largemouth bass had Hg concentrations >0.3 ppm versus Se:Hg molar ratios <1:1, respectively. These individual findings suggest that either the USEPA 0.3-ppm tissue Hg threshold is not adequately protective of all human health for some fish, or the 1:1 molar ratio is unnecessarily conservative for some fish. For example, in a situation in which Hg is below the USEPA criterion and Se is also low, a molar ratio of <1:1 may not indicate an elevated risk of Hg toxicity to the consumer. The discrepancy illuminates the need for continued study of the role of Se in Hg toxicity for the purposes of fish consumption guidance.

### 4.4. HBV_Se_ Determination

According to Ralston et al. [32], a HBV_Se_ > 0 is protective of human health (specifically the health of a child in utero), while a HBV_Se_ < 0 is not protective. The measure is based on the assumption that Hg stoichiometrically complexes with Se; as this is not the case, the HBV_Se_ is a highly conservative estimate of incurred risk from fish consumption [32]. The HBV_Se_ values from our study suggest that bluegill and crappie from Jordan Lake and bluegill from Lake Michie contain sufficiently high levels of Se to provide a measure of protection to developing embryos and young children undergoing neurodevelopment from the Hg contained in the fish, but that largemouth bass from Jordan Lake and crappie and largemouth bass from Lake Michie do not. North Carolina does not have a creel limit for bluegill [53], which is considered a low-Hg fish that is safe for up to two 170-g meals per week for sensitive populations and up to four 170-g meals per week for everyone else [22]. All crappie sampled from Jordan Lake had values >0, suggesting adequate Se to offer a measure of protection against Hg when consumed, but only 6 of 14 crappie sampled from Lake Michie had values >0, suggesting that more than half of the crappie caught from Lake Michie may pose an increased risk to sensitive subpopulations due to a deficit of Se compared to Hg. Furthermore, 6 of the 14 crappie sampled from Lake Michie had tissue Hg concentrations that exceeded the 0.3-ppm USEPA threshold. Given that both the HBV_Se_ values and the tissue Hg data from crappie from Lake Michie show that approximately half of the crappie may pose a risk for Hg toxicity to consumers, the absence of a fish consumption advisory for this species at this reservoir may warrant consideration by those issuing fish consumption advisories. Of the largemouth bass sampled, 8 of 15 from Jordan Lake and 1 of 16 from Lake Michie had values >0; this suggests that approximately half of the largemouth bass from Jordan Lake and almost none from Lake Michie are safe for consumption by all human populations. Based on these values, the statewide Hg advisory for largemouth bass is adequately protective at both reservoirs.

The number of individuals of each species that met the criteria for safe consumption was the same for both the Se:Hg molar ratio >1:1 threshold and the HBV_Se_ > 0 threshold. Further examination of these thresholds in concert may provide much needed insight to the accuracy of each and ultimately help inform risk assessment.

## 5. Conclusions

Previous studies have determined that fish tissue Hg concentrations alone are not sufficient to predict toxic outcomes in organisms [28,71]. Though the exact mechanism has not been completely characterized, the concurrent presence of Se with Hg has exhibited a mitigating effect on Hg toxicity [24,70]. Because current human health guidelines are based on tissue Hg alone, the need to more fully understand the relationship between Se and Hg and its potential role in toxicity is great. In this study, we used two approaches to quantify and examine this relationship: the Se:Hg molar ratio and the HBV_Se_. Our data showed that the number of individual fish considered safe or unsafe for consumption was the same using both the Se:Hg molar ratio and the HBV_Se_, and that these measures were more conservative than the USEPA 0.3-ppm fish tissue residue criterion for Hg alone. This was an interesting finding, as Se reduces or prevents Hg toxicity, and one would expect any measure that accounts for both Hg and Se to show more fish safe for consumption than measures using the Hg criterion alone. The Se:Hg molar ratio and the HBV_Se_ are not currently utilized for fish consumption guidance, as previously reported molar ratios show variation by species, region, and water body [25,27,28]. Our data were also variable, with the Se:Hg molar ratio as a function of total length only achieving statistical significance (*p <* 0.0001) for largemouth bass. The molar ratio and the HBV_Se_ supported the existing statewide fish consumption advisory for largemouth bass from waters in North Carolina, USA. Unexpectedly, we found that several crappie from Lake Michie, which are not under an advisory, were not safe for consumption (by sensitive subpopulations, specifically) using the USEPA Hg criterion, as well as the Se:Hg molar ratio and the HBV_Se_. We also found tissue Hg in some fish from Jordan Lake and Lake Michie to be at levels previously shown to have adverse impacts on piscivorous organisms and fish themselves. No remedial efforts are currently in place at these reservoirs, though organisms within their ecosystems may be at risk.

In order to determine the best approach to modifying recommendations related to fish consumption and regulations related to freshwater ecosystem health, the continued study of Se and Hg together in fish and other aquatic or piscivorous wildlife is necessary, whether it is by examination of the Se:Hg molar ratio, the HBV_Se_, or some other metric. Prior research has demonstrated that Hg and Se are present at different concentrations in different organs and tissues [11]; therefore, this is a consideration to enhance future studies. Other parameters for consideration in future research that may further clarify the Se:Hg relationship in freshwater fish include seasonal variation; organism age; abiotic characteristics of water, soil, and sediments; and the antagonistic or synergistic behaviors of other contaminants that commonly occur with Hg and Se. Continued efforts are needed to determine the most accurate recommendations for fish consumption to maximize the health benefits of this nutritious food source, especially for expectant mothers and young children.

## Figures and Tables

**Table 1:** Summary of freshwater fish consumption recommendations by population subgroup for Hg and Se measured in ppm wet weight (WW) in North Carolina, USA, at the time of fish collection in 2014 and 2016 [23].

Contaminant	Concentration (ppm WW)	Fish Consumption Recommendations by Population Subgroup	
		Women 15–44 Years Old (Childbearing Age) and Children < 15 Years Old	General Public (Males ≥ 15 and Females > 44 Years Old)
Hg	<0.4	2 meals/week	4 meals/week
	0.4–1.0	do not eat	1 meal/week
	>1.0–3.0	do not eat	1 meal/month
	>3.0	do not eat	do not eat
Se	<10	no advisory	no advisory
	10–20	1 meal/week	1 meal/week
	>20–50	1 meal/month	1 meal/month
	>50	do not eat	do not eat

**Table 2:** Size classification parameters for bluegill, crappie, and largemouth bass collected from Jordan Lake and Lake Michie, North Carolina, USA.

Species	Small	Medium	Large
Bluegill	<115 mm	115–150 mm	>150 mm
Crappie	<203 mm	203–279 mm	>279 mm
Largemouth bass	<355 mm	355–432 mm	>432 mm

**Table 3:** Sample size (*N*), concentration or molar ratio mean ± standard error (SE), concentration or molar ratio range, and mean total length (TL) and range (in parentheses) for three sportfish species from two reservoirs in central North Carolina, USA. Also shown are analysis of variance results (ANOVA; α = 0.05, *F*-ratio, and *p*-value) to determine the effect of TL on measured fish tissue mercury (Hg), selenium (Se), and Se:Hg molar ratio (Se:Hg); all data were log_e_-transformed to comply with a normal-distribution assumption.

Species	Bluegill		Crappie		Largemouth Bass	
Lake	Jordan	Michie	Jordan	Michie	Jordan	Michie
**Hg (ppm, wet weight)**						
*N*	30	27	24	24	31	31
Mean ± SE	0.05 ± <0.01	0.18 ± 0.01	0.07 ± 0.01	0.29 ± 0.03	0.28 ± 0.03	0.71 ± 0.06
Range	0.01–0.12	0.08–0.41	0.03–0.15	0.12–0.58	0.04–0.68	0.23–1.66
Mean TL	128	135	249	223	365	382
(mm, and range)	(75–178)	(104–265)	(140–300)	(145–300)	(200–531)	(236–545)
ANOVA Hg vs. TL, *F*-Ratio	1.1432	0.0167	11.3894	4.5751	41.3917	41.3247
ANOVA Hg vs. TL, *p*-Value	0.2941	0.8981	0.0027	0.0438	<0.0001	<0.0001
**Se (ppm, wet weight)**						
*N*	15	12	10	14	15	16
Mean ± SE	0.10 ± 0.01	0.15 ± 0.01	0.10 ± 0.01	0.10 ± <0.01	0.09 ± <0.01	0.08 ± <0.01
Range	0.01–0.13	0.08–0.21	0.07–0.13	0.08–0.11	0.07–0.12	0.07–0.10
Mean TL	132	135	259	233	376	379
(mm, and range)	(75–177)	(107–265)	(211–300)	(189–300)	(208–531)	(236–545)
ANOVA Se vs. TL, *F*-Ratio	12.8870	13.3951	0.3572	5.3774	8.1846	1.2667
ANOVA Se vs. TL, *p*-Value	0.0033	0.0044	0.5666	0.0388	0.0134	0.2793
**Se:Hg Molar Ratio**						
*N*	15	12	10	14	15	16
Mean ± SE	6.18 ± 0.59	2.70 ± 0.40	5.67 ± 0.72	1.04 ± 0.13	1.67 ± 0.37	0.44 ± 0.07
Range	2.17–11.50	1.25–5.23	3.30–9.36	0.51–1.98	0.40–4.82	0.16–1.04
Mean TL	132	135	259	233	376	379
(mm, and range)	(75–177)	(107–265)	(211–300)	(189–300)	(208–531)	(236–545)
ANOVA Se:Hg vs. TL, *F*-Ratio	2.0049	3.7044	11.3559	2.3819	68.9019	48.4018
ANOVA Se:Hg vs. TL, *p*-Value	0.1803	0.0832	0.0098	0.1487	<0.0001	<0.0001

**Table 4:** Sample size (*N*), Se health benefit value (HBV_Se_) mean ± standard error (SE), and range for three sportfish species from two reservoirs in central North Carolina, USA.

Lake	Species	*N*	HBV_Se_ Mean ± SE	HBV_Se_ Range
Jordan				
	Bluegill	15	1.24 ± 0.11	0.11–1.58
	Crappie	10	1.24 ± 0.07	0.90–1.63
	Largemouth Bass	15	−0.85 ± 0.62	−6.50–1.03
Michie				
	Bluegill	12	1.46 ± 0.18	0.36–2.49
	Crappie	14	−0.62 ± 0.40	−3.88–0.99
	Largemouth Bass	16	−12.03 ± 3.53	−51.90–0.10
